# Ultrasound-based nomogram to predict the recurrence in papillary thyroid carcinoma using machine learning

**DOI:** 10.1186/s12885-024-12546-6

**Published:** 2024-07-07

**Authors:** Binqian Zhou, Jianxin Liu, Yaqin Yang, Xuewei Ye, Yang Liu, Mingfeng Mao, Xiaofeng Sun, Xinwu Cui, Qin Zhou

**Affiliations:** 1 Department of Ultrasound, The Central Hospital of Wuhan, Tongji Medical College, Huazhong University of Science and Technology, Wuhan, 430014 China; 2 Department of Medical Ultrasound, Tongji Hospital, Tongji Medical College, Huazhong University of Science and Technology, Wuhan, 430014 China

**Keywords:** Papillary thyroid carcinoma, Recurrence, Radiomics, Nomogram, Ultrasound

## Abstract

**Background and aims:**

The recurrence of papillary thyroid carcinoma (PTC) is not unusual and associated with risk of death. This study is aimed to construct a nomogram that combines clinicopathological characteristics and ultrasound radiomics signatures to predict the recurrence in PTC.

**Methods:**

A total of 554 patients with PTC who underwent ultrasound imaging before total thyroidectomy were included. Among them, 79 experienced at least one recurrence. Then 388 were divided into the training cohort and 166 into the validation cohort. The radiomics features were extracted from the region of interest (ROI) we manually drew on the tumor image. The feature selection was conducted using Cox regression and least absolute shrinkage and selection operator (LASSO) analysis. And multivariate Cox regression analysis was used to build the combined nomogram using radiomics signatures and significant clinicopathological characteristics. The efficiency of the nomogram was evaluated by receiver operating characteristic (ROC) curves, calibration curves and decision curve analysis (DCA). Kaplan-Meier analysis was used to analyze the recurrence-free survival (RFS) in different radiomics scores (Rad-scores) and risk scores.

**Results:**

The combined nomogram demonstrated the best performance and achieved an area under the curve (AUC) of 0.851 (95% CI: 0.788 to 0.913) in comparison to that of the radiomics signature and the clinical model in the training cohort at 3 years. In the validation cohort, the combined nomogram (AUC = 0.885, 95% CI: 0.805 to 0.930) also performed better. The calibration curves and DCA verified the clinical usefulness of combined nomogram. And the Kaplan-Meier analysis showed that in the training cohort, the cumulative RFS in patients with higher Rad-score was significantly lower than that in patients with lower Rad-score (92.0% vs. 71.9%, log rank *P* < 0.001), and the cumulative RFS in patients with higher risk score was significantly lower than that in patients with lower risk score (97.5% vs. 73.5%, log rank *P* < 0.001). In the validation cohort, patients with a higher Rad-score and a higher risk score also had a significantly lower RFS.

**Conclusion:**

We proposed a nomogram combining clinicopathological variables and ultrasound radiomics signatures with excellent performance for recurrence prediction in PTC patients.

## Introduction

Thyroid cancer has grown to be one of the most common cancers worldwide, and papillary thyroid cancer (PTC) accounts for the majority [1]. Thanks to the surgical approach and following radioactive iodine (RAI) therapy recommended by several Guidelines [2–5], there is a 90–95% long-term survival in the overall population with PTC. Nevertheless, there still exists a small portion of patients who would eventually develop either local cervical recurrences or even distant metastases to the lung, bone, and liver [6]. And those patients would have no choice but to undergo secondary surgery or high dose RAI therapy, which in turn poses a threat to both patients’ physical and mental health, let alone for those who are not suitable candidates for another surgery. Accordingly, early identification of possible recurrent malignant nodules is rather important when it comes to PTC.

As indicated by several previous studies [7–11], age, tumor size, extrathyroidal extension (ETE), lymph node (LN) metastases, distant metastases, and tumor-node-metastases (TNM) stage are the significantly prognostic factors in patients with PTC. In clinical practice, ultrasound (US) is now the first-line diagnostic technique that evaluates thyroid nodules and cervical LNs. And US features associated with malignant nodules are also thought to be related to the recurrence of PTC. For example, Kim SY et al. found that malignant-appearing US features and larger nodule size were independently associated with PTC recurrence [12]. And another study demonstrated that preoperative US features of LNs, including size and hyperechogenicity, may be valuable for predicting recurrence in PTC patients [13]. The above evidence inspired us that US features held great potential for predicting the prognosis of PTC. However, the features that human eye could capture are very limited and with poor repeatability, which makes it almost impossible to predict whether or not PTC would recur. Thus, we are in urgent need of an approach to capture and analyze as much US features as possible.

In the era of Internet, radiomics is a rapidly evolving field that quantifies high-throughput features from medical images and is useful in cancer screening, diagnosis, and prognosis evaluation [14, 15]. As for applications in PTC, some studies have already verified their value in diagnosis of thyroid malignancy [16–19], detection of neck metastatic LNs [20, 21], and management of thyroid nodules [22]. In some studies, the performance of radiomics even showed significantly better sensitivity, specificity and accuracy than those of radiologists [22–24].

In this study, we proposed a nomogram combining US radiomics features and clinicopathological characteristics to help predict the recurrence of PTC and hopefully would guide therapeutic regimen accordingly.

## Materials and methods

The Institutional Review Board (IRB) of our hospital approved this retrospective study (WHZXKYL2022-217), and written informed consent was waived. This study was completed in accordance with the Declaration of Helsinki as revised in 2013.

### Patients

**Fig. 1:**
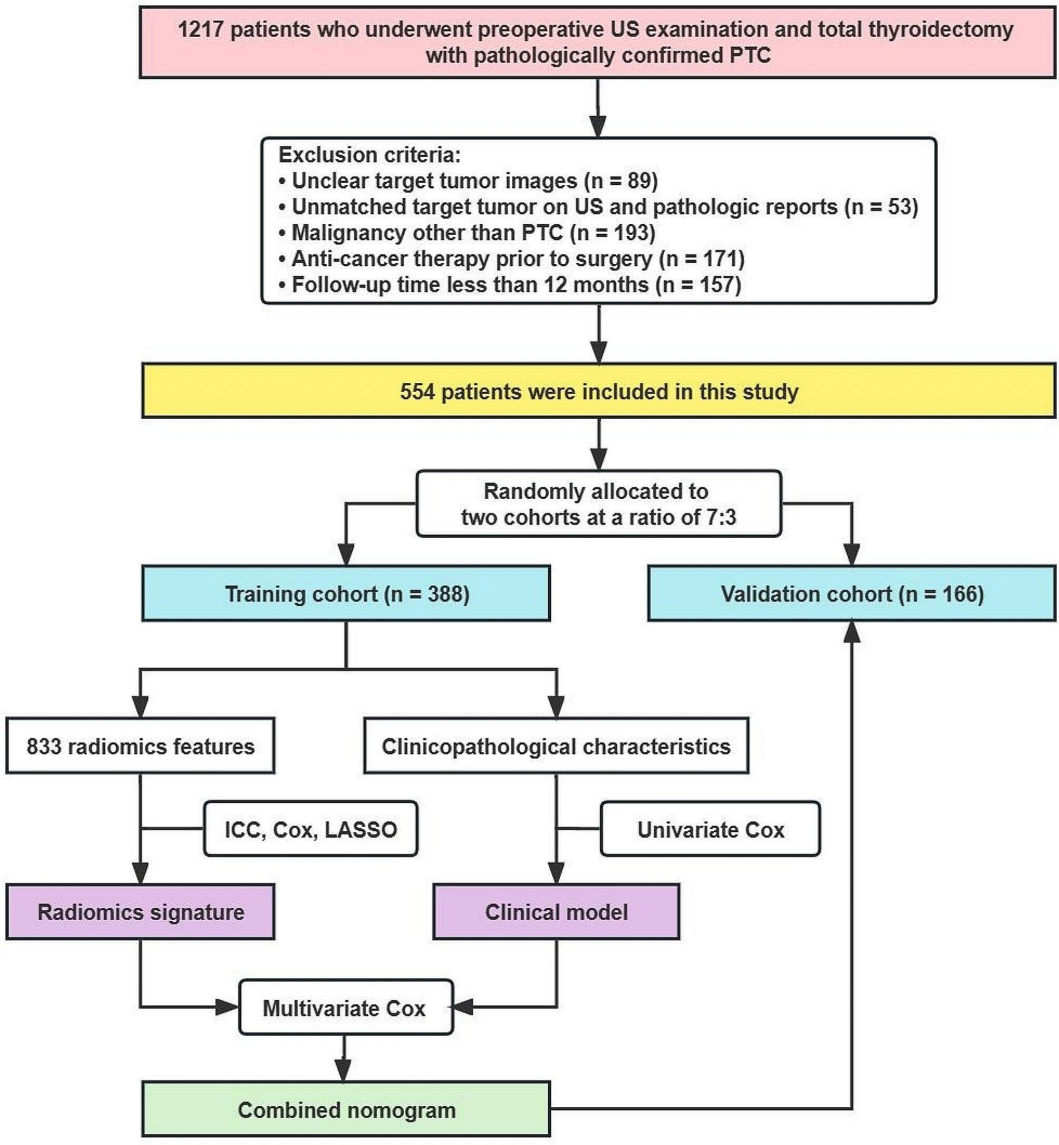
The flowchart of this study. US, ultrasound; PTC, papillary thyroid carcinoma; ICC, intraclass correlation coefficient; LASSO, least absolute shrinkage and selection operator

Patients who underwent preoperative US examination and total thyroidectomy were collected from the institutional database between July 2017 and August 2021. The inclusion criteria were as follows: (a) pathologically confirmed PTC; (b) available preoperative US images within 2 weeks before surgery; (c) complete follow-up records. The exclusion criteria were as follows: (a) unclear target tumor images due to artifacts; (b) unmatched tumor size and location on US image and pathologic reports; (c) diagnosed with malignancy other than PTC; (d) any anti-cancer therapy prior to surgery, such as chemotherapy, radiotherapy, or immunotherapy; (e) follow-up time less than 12 months after surgery. The study flowchart is shown in Fig. 1.

### Data collection

Clinical information including age, sex, and preoperative serology examination results including thyroid-stimulating hormone (TSH), free thyroxine (FT), thyroglobulin (Tg), anti-Tg antibody (TgAb) and thyroid peroxidase antibody (TpoAb) were acquired from medical records.

For included patients, total thyroidectomy with routinely prophylactic central LN dissection was performed. Lateral compartment LN dissection was also performed when LN metastasis was diagnosed at preoperative US-guided fine needle aspiration (FNA) or intraoperative frozen section.

Tumor size, laterality, multifocality, capsular invasion, ETE, LN metastasis status, Hashimoto thyroiditis and nodular goiter were recorded according to the original pathologic reports.

### US image acquisition and US-reported tumor morphologic features

The US images were acquired using US equipments with 5–12 MHz linear array transducer (EPIQ 7 and iU22; Philips Medical Systems, the Netherlands), 6–18 MHz linear array transducer (Acuson Oxana1; Simens Healthcare, Germany) and 6–15 MHz linear array transducer (GE Logiq E8; GE Healthcare, the United States).

The clinical US diagnoses were performed according to standard protocols [25] and the American College of Radiology Thyroid Imaging, Reporting and Data System (TI-RADS) [26]. For each case, one or two most representative images which displayed the target tumor in the longest axis cross section were selected. And the tumors’ important morphologic US features, such as upper/lower pole of thyroid gland, left/right lobe or isthmus, solid or mixed cystic and solid and TI-RADS level were re-assessed and verified by two radiologists (B.Z. and Y.Y., with 5–10 years of experience in thyroid imaging) according to the original images. Any disagreement was resolved through consulting a senior radiologist (J.L., with 20 years of experience in thyroid imaging). All observers were blinded to the clinical data and pathologic results.

### Postoperative follow-up and recurrence

After surgery, all patients received TSH suppression treatment and RAI therapy with 50–200 mCi. And they were followed up every 6 months with a neck US, a chest computed tomography (CT), and laboratory examinations of Tg and TgAb. Moreover, if serum Tg or TgAb were detectable with no suspicious evidence on neck US or chest CT scan, iodine 131 whole-body scintigraphy or fluorodeoxyglucose positron emission tomography (PET)/CT were further performed to determine recurrence.

According to 2015 American Thyroid Association (ATA) Guidelines [2], incomplete response was defined as negative imaging and suppressed Tg ≥ 1 ng/mL or stimulated Tg ≥ 10 ng/mL or rising anti-Tg antibody levels / structural or functional evidence of disease with any Tg level with or without anti-Tg antibodies. Recurrence was defined as incomplete response after any period with no evidence of disease. The end point was recurrence-free survival (RFS), which was defined as the period from the date of the surgery to the date of the first recurrence (biochemical, structural, or functional) or to the last follow-up visit.

### Region of interest segmentation and radiomics feature extraction

**Fig. 2:**
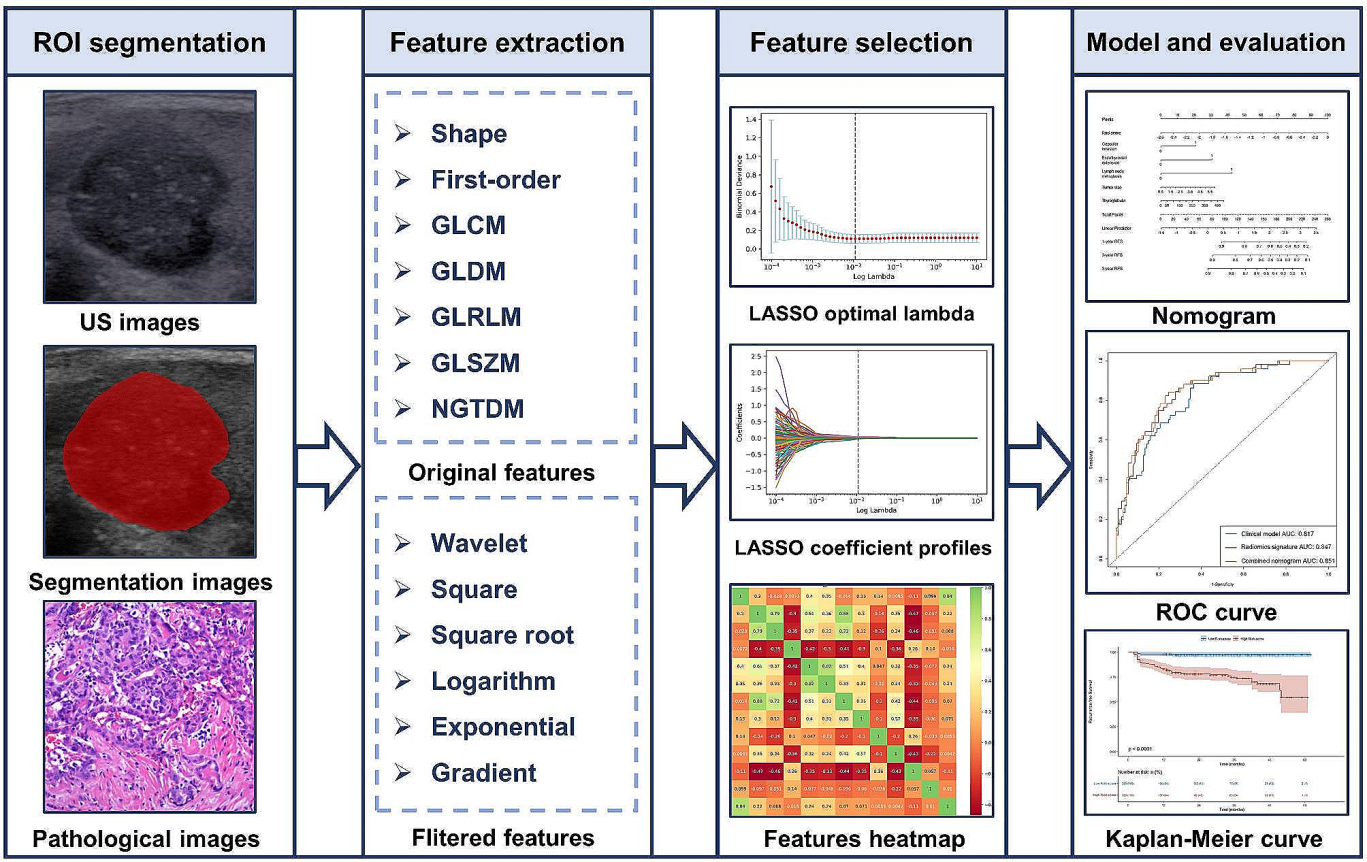
The flowchart of radiomics study. ROI, region of interest; US, ultrasound; LASSO, least absolute shrinkage and selection operator; ROC, receiver operating characteristic

The workflow of radiomics analysis was shown in Fig. 2. For region of interest (ROI) segmentation, we previously normalized each image for comparison between patients using different equipments. The ROIs were manually delineated by drawing the tumor contour on the ultrasound image using ITK-SNAP software. All radiologists (B.Z. and X.Y., responsible for drawing, and M.M., responsible for redrawing when disagreements occured) were blinded to patients’ information. Then features were extracted using an open-source software (Pyradiomics). Subsequently, to assess the interobserver and intraobserver agreement of the feature extraction, 50 patients were randomly selected for retest and the intraclass correlation coefficient (ICC) was calculated. Finally, features with an ICC of < 0.8 were excluded for the following analysis.

### Feature selection and radiomics signature construction

First, the correlation among features was evaluated by Pearson’s correlation test. And the univariate Cox proportional hazards model was applied to identify features highly related to recurrence (*P* < 0.05) in the training cohort. Then the radiomics signature was constructed using the most predictive recurrence-related features selected by the least absolute shrinkage and selection operator (LASSO) regression method with ten-fold cross-validation. LASSO was selected for its effeciency in handling high-dimensional data and its capability to perform feature selection by shrinking less important feature coefficients to zero [27]. For each patient, we also developed a radiomics score (Rad-score) through a linear combination of selected features weighted by their respective coefficients. And the cut-off value of Rad-score was calculated according to the Youden index in training cohort.

### Clinical model and combined nomogram construction

In the training cohort, significant clinicopathological prognostic factors were selected as potential factors using the univariate Cox regression. Then, all potential factors (*P* < 0.05) were introduced into the multivariate Cox regression model to build the clinical model.

Consequently, we incorporated the Rad-score and significant clinicopathological variables to build the combined nomogram in the training cohort. According to Royston. et al. [28], we determined Harrell’s C-index, Gönen & Heller K index and the explained variation on the log relative hazard scale based on the D statistic (R^2^_D_) as a measure of model discrimination. For each patient, risk score was also calculated from the combined nomogram. And the cut-off value of risk score was calculated according to the Youden index. For calibration of the combined nomogram, a calibration curve was drawn. And the decision curve analysis (DCA) was used to quantify the net benefits from the three models at different threshold probabilities in the trainning and validation cohorts [29].

### Performance of the combined nomogram

The prognostic performance of clinical model, radiomics signature and combined nomogram for 1, 2 and 3 years RFS were assessed using receiver-operating characteristic (ROC) analysis. And the area under the curve (AUC) was evaluated for the prognostic efficiency of the three models [30]. The Delong test was used to compare different AUC.

### Recurrence-free survival analysis

Survival analysis was performed to explore the potential of the radiomics signature and combined nomogram to predict RFS in PTC patients. For each patient in the training and validation cohorts, the Rad-score and risk score were divided into high score group and low score group according to the threshold calculated from the Youden index in the training cohort. The Kaplan-Meier curve and log-rank test were used to analyze the RFS of patients with different Rad-scores and risk scores.

### Statistical analysis

Statistical analysis was performed using R (version 4.2.2), IBM SPSS (version 22.0) and Python (version 3.7) softwares. Continuous variables were expressed as mean ± standard deviation (SD) or median (interquartile range [IQR]), and the differences were assessed using the t-test or Mann-Whitney U test. Categorical variables were presented as total and percentage, and the differences were compared using the chi-square test or Fisher exact test. LASSO regression and Cox regression were built using the “glmnet” and “survival” R packages, respectively. Kaplan–Meier curves were constructed by using the R packages named “survival” and “survminer”. *P* < 0.05 was considered statistically significant.

## Results

### Baseline characteristics

A total of 554 patients (111 men and 443 women; mean age, 50.7 ± 11.7 years) were retrospectively included in our final study cohort, and all patients underwent total thyroidectomy and central neck dissection. The median follow-up time was 23 months (range 12–62 months). And during the follow up, the total recurrence event was 79. Then all patients were randomly allocated to the training (*n* = 388) and validation (*n* = 166) cohort at a ratio of 7:3, and the recurrence event was respectively 54 and 25. The patients’ clinical and pathological characteristics were summarized in Table 1. As demonstrated, some pathological characteristics (tumor size, capsular invasion, ETE, LN metastasis) and one laboratory parameter (preoperative Tg) were significantly different between patients with or without recurrence in the training and validation cohorts.

**Table 1:** Baseline characteristics of patients in the training and validation cohorts

**Characteristics**	Training cohort (*n* = 388)			**Validation cohort (*n*** **= 166)**		
	Recurrence **(*n*** **= 54)**	Non-recurrence (*n* = 334)	*P* values	Recurrence (*n* = 25)	Non-recurrence (*n* = 141)	*P* values
**Demographic Characteristics**						
Age, years	49.6 ± 12.0	51.6 ± 11.6	0.227	46.5 ± 12.8	49.8 ± 11.3	0.193
Gender, male	6 (11.1)	64 (19.2)	0.184	8 (32.0)	33 (23.4)	0.450
**US characteristics**						
Tumor location			0.239			0.317
upper pole	31 (57.4)	177 (53.0)		13 (52.0)	82 (58.2)	
lower pole	23 (42.6)	157 (47.0)		12 (48.0)	59 (41.8)	
Primary site			0.600			1.000
Left/Right lobe	54 (100)	327 (97.9)		24 (96.0)	136 (96.5)	
Isthmus	0 (0)	7 (2.1)		1 (4.0)	5 (3.5)	
Composition			0.695			0.671
Solid	53 (98.1)	330 (98.8)		24 (96.0)	141 (100)	
Mixed cystic and solid	1 (1.9)	4 (1.2)		1 (4.0)	0 (0)	
TI-RADS level			0.348			0.262
TR 4	29 (53.7)	178 (53.3)		14 (56.0)	67 (47.5)	
TR 5	25 (46.3)	156 (46.7)		11 (44.0)	74 (52.5)	
**Pathological characteristics**						
Tumor size, mm	19.3 ± 11.3	10.9 ± 6.8	< 0.001	21.2 ± 12.5	11.0 ± 6.6	< 0.001
Laterality			0.464			0.665
Unilateral	23 (42.6)	163 (48.8)		10 (40.0)	65 (46.1)	
Bilateral	31 (57.4)	171 (51.2)		15 (60.0)	76 (53.9)	
Multifocality	35 (64.8)	191 (57.2)	0.303	15 (60.0)	87 (61.7)	1.000
Capsular invasion	42 (77.8)	175 (52.4)	0.001	22 (88.0)	71 (50.4)	0.001
Extrathyroidal extension	8 (14.8)	16 (4.8)	0.010	4 (16.0)	7 (5.0)	0.034
Lymph node metastasis	44 (81.5)	175 (52.4)	< 0.001	22 (88.0)	71 (50.4)	0.001
Hashimoto thyroiditis	7 (13.0)	65 (19.5)	0.269	2 (8.0)	26 (18.4)	0.257
Nodular goiter	36 (66.7)	258 (77.2)	0.122	16 (64.0)	107 (75.9)	0.222
**Laboratory parameters**						
TSH, µIU/mL	1.8 (1.2–2.6)	1.7 (1.1–2.5)	0.564	1.7 (1.3–2.6)	1.9 (1.3–2.6)	0.515
FT3, pmol/L	4.6 (4.1-5.0)	4.6 (4.1–5.1)	0.761	4.8 (4.5–5.5)	4.7 (4.4–5.3)	0.696
FT4, pmol/L	12.9 (11.6–14.1)	12.2 (10.7–13.7)	0.132	12.2 (11.4–13.6)	12.3 (11.2–13.8)	0.981
Tg, ng/mL	24.3 (10.7-144.6)	21.7 (8.5–71.4)	0.037	23.7 (9.2–82.0)	22.0 (8.9–72.9)	0.045
TgAb, IU/mL	1.6 (0.2–7.1)	1.5 (0.4–19.2)	0.518	0.7 (0.1–1.7)	1.4 (0.1–26.5)	0.143
TpoAb, IU/mL	0.5 (0.3–1.5)	0.7 (0.3–12.3)	0.515	0.3 (0.1–1.4)	0.6 (0.3–10.4)	0.097

Data presented as mean ± SD or number of patients (%) or median (IQR) where appropriate

Abbreviation: US, ultrasonography; TI-RADS, Thyroid Imaging, Reporting and Data System; TSH, thyroid-stimulating hormone; FT, free thyroxine; Tg, thyroglobulin; TgAb, thyroglobulin antibody; TpoAb, thyroid peroxidase antibody

### Feature selection and radiomics signature establishment

**Fig. 3:**
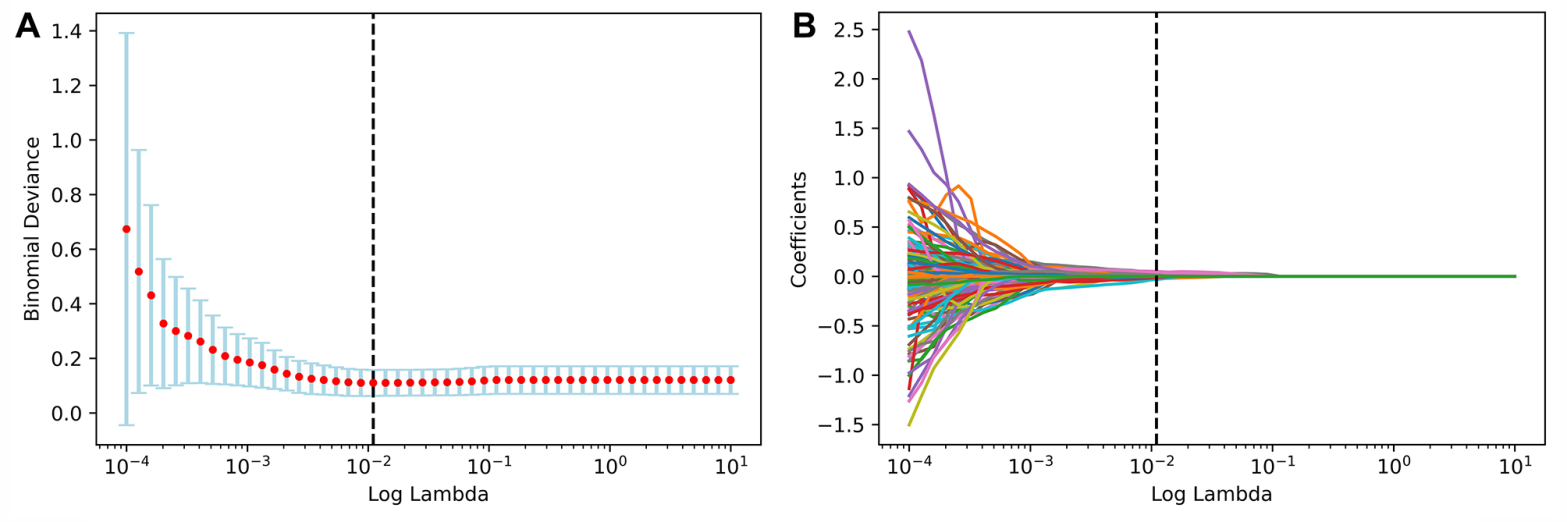
Radiomics feature selection by LASSO regression. **(A)** Selection of optimal lambda value using ten-fold cross-validation by the minimum criteria. **(B)** LASSO coefficient profiles of the radiomics features. LASSO, least absolute shrinkage and selection operator

For each patient, only one image of the largest nodule was used. After pyradiomics, 833 imaging features were extracted from each image. Then the features were selected and reduced to 13 recurrence-related features after ICC test, Cox regression and LASSO algorithm in the training cohort (Fig. 3). The optimal lambda value for the LASSO regression was determiend using ten-fold cross-validation based on the minimum criteria. LASSO selected features and their coefficients were shown in Table 2. Finally, the Rad-score was calculated for each patient.

**Table 2:** LASSO selected features and their coefficients

Features	Coefficient values
original_glrlm_RunVariance	0.029335
original_glszm_GrayLevelNonUniformity	0.013971
original_glszm_SizeZoneNonUniformity	0.010112
wavelet.LLH_firstorder_Minimum	-0.013079
wavelet.LLH_glrlm_RunLengthNonUniformityNormalized	0.009000
wavelet.LLH_glrlm_ShortRunHighGrayLevelEmphasis	0.020514
wavelet.LHL_glszm_GrayLevelNonUniformity	0.045947
wavelet.LHH_glszm_ZoneEntropy	0.000113
wavelet.HLL_ngtdm_Contrast	-0.010821
wavelet.HLH_glszm_ZoneEntropy	0.010673
wavelet.HLH_glszm_ZonePercentage	-0.000557
wavelet.HHL_glcm_ClusterProminence	0.023622
wavelet.LLL_glszm_ZoneVariance	0.003228

**Fig. 4:**
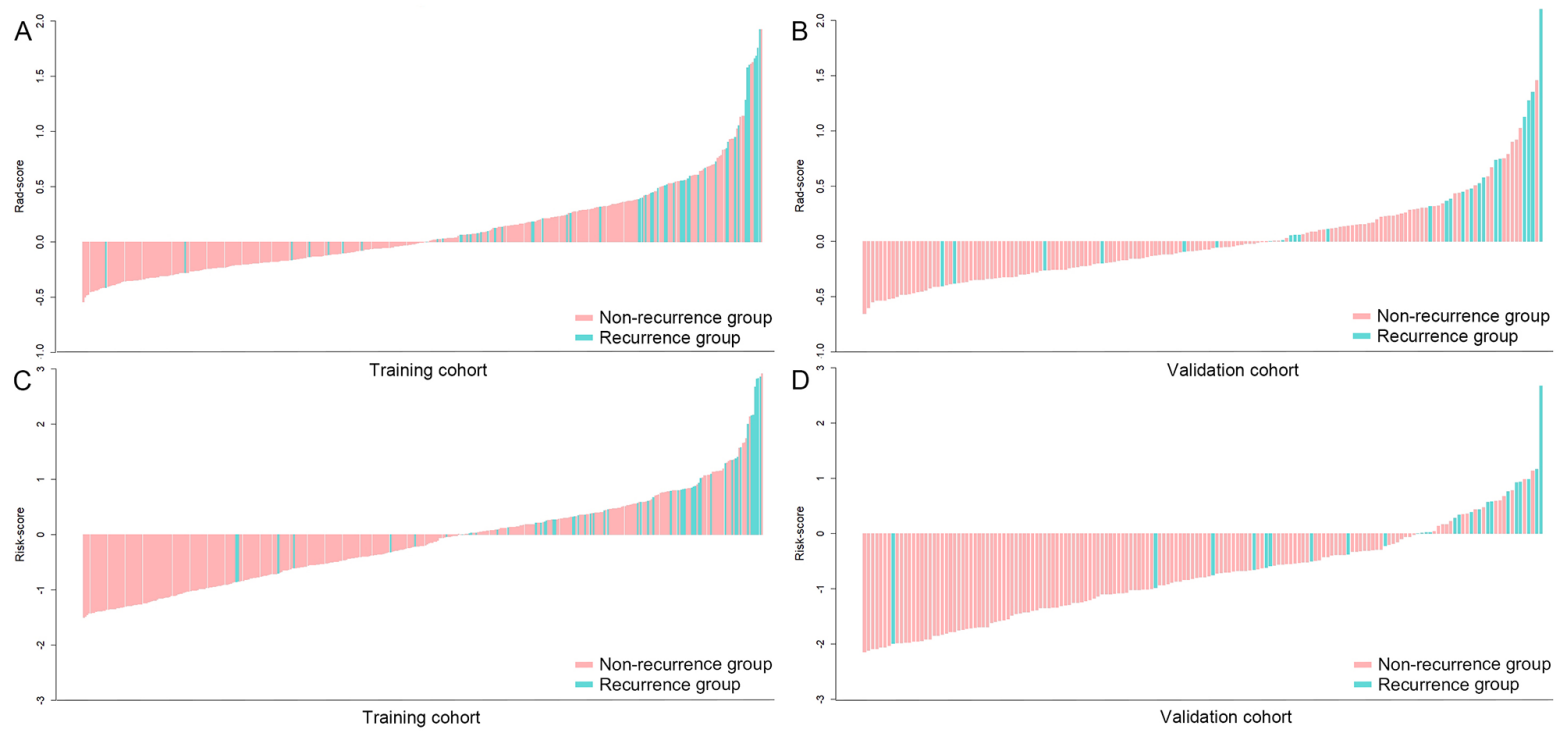
Rad-scores from radiomics signature and risk scores from combined nomogram. Rad-score of each patient in the training **(A)** and validation **(B)** cohorts. Risk score of each patient in the training **(C)** and validation **(D)** cohorts. Rad-score, radiomics score

Then the Rad-score bar plot for each patient was plotted using the cut-off value in the training (Fig. 4A) and validation (Fig. 4B) cohorts.

### Clinical model establishment

According to the univariate Cox analysis in the training cohort, capsular invasion (HR = 2.824, 95%CI = 1.486–5.365, *P* = 0.002), tumor size (HR = 1.062, 95%CI = 1.044–1.081, *P* < 0.001), ETE (HR = 3.335, 95%CI = 1.565–7.104, *P* = 0.002), LN metastasis (HR = 3.553, 95%CI = 1.787–7.064, *P* < 0.001), preoperative Tg (HR = 1.004, 95%CI = 1.002–1.006, *P* < 0.001) were identified as risk factors for recurrence in PTC patients. We incorporated the above clinical variables into the multivariate Cox regression model to construct a clinical prediction model.

### Development and validation of combined nomogram

The univariate and multivariate Cox regression analysis including Rad-score and the clinicopathological variables were performed, and the results were shown in Table 3. The Rad-score (HR = 3.790, 95%CI = 2.468–5.819, *P* < 0.001) and LN metastasis (HR = 2.518, 95%CI = 1.246–5.088, *P* = 0.010) were identified as independent predictors for recurrence. The Harrell’s C-index, Gönen & Heller K index and the explained variation-R^2^_D_ for RFS were repectively 0.846 (0.017), 0.822 (0.016), 0.179 (0.053), thus the model showed a good discrimination performance.

**Table 3:** Univariate and multivariate Cox regression analysis of factors associated with RFS in the training cohort

Variables	Univariate analysis			Multivariate analysis		
	β	HR (95% CI)	*P* values	β	HR (95% CI)	*P* values
Age, years	-0.012	0.988 (0.966–1.011)	0.300	-	-	-
Gender, male	0.643	0.526 (0.226–1.235)	0.138	-	-	-
Tumor size, mm	0.061	1.062 (1.044–1.081)	< 0.001	-	-	-
Capsular invasion	1.038	2.824 (1.486–5.365)	0.002	-	-	-
Extrathyroidal extension	1.205	3.335 (1.565–7.104)	0.002	-	-	-
Lymph node metastasis	1.268	3.553 (1.787–7.064)	< 0.001	0.924	2.518 (1.246–5.088)	0.010
Tg, ng/mL	0.004	1.004 (1.002–1.006)	< 0.001	-	-	-
Rad-score	1.496	4.463 (2.953–6.745)	< 0.001	1.332	3.790 (2.468–5.819)	< 0.001

Abbreviation: HR, hazard ratio; CI, confidence internal; Tg, thyroglobulin

**Fig. 5:**
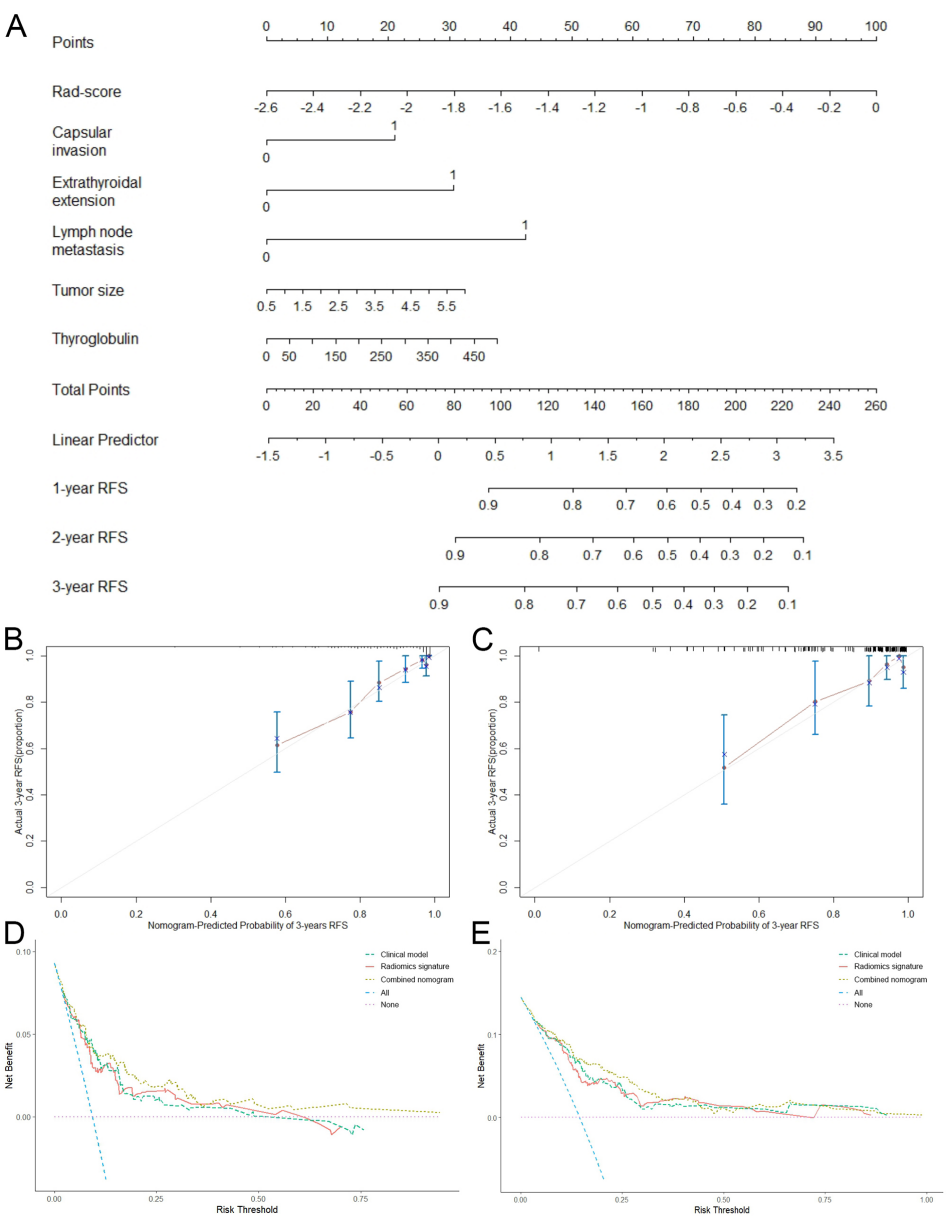
The validation of combined nomogram. **(A)** Combined nomogram based on clinicopathological characteristics and radiomics signature. Calibration curves of the combined nomogram at 3-year RFS in the training **(B)** and validation **(C)** cohorts. Decision curves of the clinical model, radiomics signature and combined nomogram at 3-year RFS in the training **(D)** and validation **(E)** cohorts. Rad-score, radiomics score; RFS, recurrence-free survival

Then we developed a predictive combined nomogram, which was shown in Fig. 5A. As demonstrated by the calibration curve (Fig. 5B and C) at 3 years, the combined nomogram showed good agreement between the predictive probability and actual events of recurrence, which suggested it was a perfect fit. The DCA of three models in the training and validation cohorts at 3 years were shown in Fig. 5D and E. As demonstrated, both the radiomics signature and combined nomogram yielded better net benefit to predict recurrence event than the clinical model at most of the threshold probabilities.

### Performance of combined nomogram

**Fig. 6:**
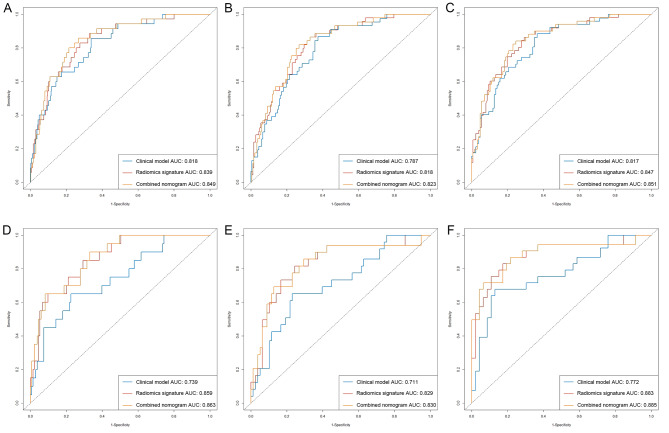
Performance of the clinical model, radiomics signature and combined nomogram. ROC curves of three models at 1, 2 and 3 years in the training (**A**, **B** and **C**) and validation (**D**, **E** and **F**) cohorts. ROC, receiver operating characteristic; AUC, area under the curve

To evaluate the performance of combined nomogram, we constructed ROC curves of three models in the training (Fig. 6A, B, C) and validation cohorts (Fig. 6D, E, F) at 1, 2 and 3 years. As demonstrated by ROC curves at 3 years, the nomogram achieved the best discrimination effect between recurrence group and non-recurrence group, with an AUC of 0.851 (95% CI: 0.788 to 0.913) in comparison to that of the radiomics signature (AUC = 0.847, 95% CI: 0.716 to 0.905) and the clinical model (AUC = 0.817, 95% CI: 0.734 to 0.894) in the training cohort. In the validation cohort, the combined nomogram (AUC = 0.885, 95% CI: 0.805 to 0.930) also exhibited better prediction of recurrence than the radiomics signature (AUC = 0.883, 95% CI: 0.801 to 0.922) and the clinical model (AUC = 0.772, 95% CI: 0.693 to 0.869). The AUC, sensitivity, specificity and accuracy of three models at 3 years were shown in Table 4. And the performance comparison among the three models were shown in Table 5.

**Table 4:** Model performance on predicting 3-year RFS probability

Models	Cohorts	AUC (95% CI)	Sensitivity	Specificity	Accuracy
Clinical model	Training	0.817 (0.734–0.894)	0.533	0.877	0.863
	Validation	0.772 (0.693–0.869)	0.429	0.862	0.843
Radiomics signature	Training	0.847 (0.716–0.905)	0.778	0.876	0.874
	Validation	0.883 (0.801–0.922)	0.667	0.859	0.855
Combined nomogram	Training	0.851 (0.788–0.913)	0.727	0.878	0.874
	Validation	0.885 (0.805–0.930)	0.750	0.864	0.861

Abbreviation: AUC, area under the receiver operating characteristic curve; CI, confidence internal

**Table 5:** Performance comparison on predicting 3-year RFS probability

Delong Test	Clinical model	Radiomics signature		Combined nomogram
Clinical model	/		0.462 ^b^	0.230 ^b^
Radiomics signature	0.504 ^a^		/	0.893 ^b^
Combined nomogram	0.287 ^a^		0.619 ^a^	/

^a^ Training cohort; ^b^ Validation cohort

In addition, the risk score bar plot was plotted using the cut-off value of risk score in the training (Fig. 4C) and validation (Fig. 4D) cohorts.

### Kaplan–Meier analysis based on different rad-scores and risk scores

**Fig. 7:**
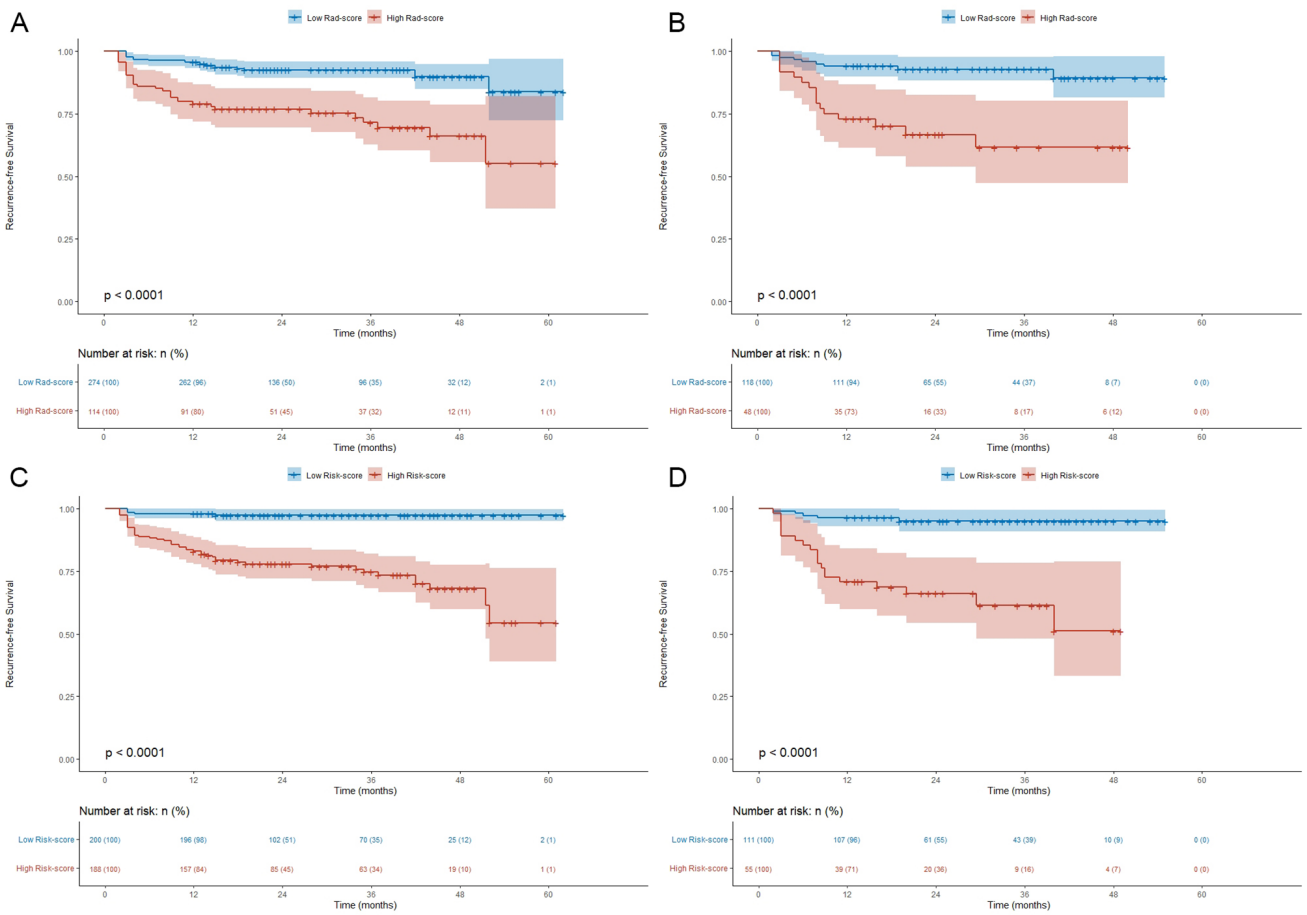
Recurrence-free survival analysis. Kaplan-Meier curves of different Rad-scores in the training **(A)** and validation **(B)** cohorts. Kaplan-Meier curves of different risk scores in the training **(C)** and validation **(D)** cohorts. Rad-score, radiomics score

In the training and validation cohorts, we divided Rad-scores and risk scores into high score group and low score group according to their cutoff values. The prognostic difference between the high score group and low score group was analyzed by Kaplan–Meier curves (Fig. 7). In the training cohort, the cumulative RFS in patients with higher Rad-score was significantly lower than that in patients with lower Rad-score (92.0% vs. 71.9%, log rank *P* < 0.001) (Fig. 7A), and the cumulative RFS in patients with higher risk score was significantly lower than that in patients with lower risk score (97.5% vs. 73.5%, log rank *P* < 0.001) (Fig. 7C). In the validation cohort, the cumulative RFS in higher Rad-score group was significantly lower than that in lower Rad-score group (92.4% vs. 66.7%, log rank *P* < 0.001) (Fig. 7B), and the cumulative RFS in higher risk score group was significantly lower than that in lower risk score group (95.5% vs. 63.6%, log rank *P* < 0.001) (Fig. 7D).

## Discussion

In this study, we evaluated the ability of a combined nomogram to estimate RFS in patients with PTC. And we came to a conclusion that the combined nomogram achieved the best discrimination effect between PTC patients with or without recurrence compared to the radiomics signature and the clinical model alone.

Although the mortality rate for PTC is low and the 5-year survival rates are high, recurrence is not unusual and is still a cause of death [31]. Several variables such as age, tumor size, ETE and LN metastasis were known risk factors for recurrence of PTC according to previous reports [32–34]. In our study, capsular invasion, tumor size, ETE, LN metastasis and preoperative Tg were identified as risk factors. As demonstrated by various studies, capsular invasion is the major determinant of clinical behavior in thyroid tumors [35–37]. And in recent studies, serum Tg was also considered a risk factor and an independent predictor for thyroid cancer [38–41]. Therefore, we have reason to believe that capsular invasion and preoperative Tg could serve as risk factors for recurrence prediction in patients with PTC.

Furthermore, we found that the Rad-score was also an independent predictor of recurrence in PTC. Recently, the US radiomics signatures have been widely used to predict many aspects of tumor behaviors in various organs [42–45]. And Park et al. found that radiomics features extracted from ultrasound images might be potential imaging biomarkers for risk stratification in patients with PTC [46]. However, no previous studies have combined clinicopathological characteristics and US radiomics signatures to predict the recurrence of PTC. In our study, we constructed a model that integrated not only radiomics signature but also significant clinicopathological parameters for recurrence prediction in PTC. And the combined nomogram outperformed clinical model and radiomics signature alone. We also performed survival analysis with regard to different Rad-scores and risk scores, and the results showed that the higher the score is, the more likely the patient is to suffer a recurrence. Therefore, the radiomics signature and the proposed nomogram both hold credible and reliable prognostic value for recurrence prediction in PTC.

In several previous studies, researcheres have also conducted predictive models for recurrence in PTC [47–49]. However, all of them only took clinico-pathologic factors or gene panels into consideration. Medical images, on the other hand, contain a vast of information and tumor features which could be read by radiomics and be further analyzed to predict some events. Xu et al. proposed an Iodine map-based radiomics model for predicting extrathyroidal extension and recurrence risk in PTC patients [50]. And although the predictive accuracy was comparable to our study, the radiation generated during computed tomography (CT) examinations could be harmful for human body, which makes it unfit for postoperative review. In contrast, ultrasound is a convenient, safe and inexpensive imaging method and is the primary choice for the evaluation of thyroid cancer. Therefore, a prognostic model based on US images may be more reasonable and accurate in the long run.

More importantly, the predictive accuracy of our nomogram offers potential clinical utility by aiding in the personalized monitoring and management strategies for patients at higher risk of recurrence. For example, if a patient was predicted with a higher risk for recurrence pre-operatively, then the surgical program would be more thoroughly especially as to the lymph node dissection. Moreover, he or she would be suggested with a more rigorous follow-up plan than those predicted with a lower risk, which could significantly improve the efficiency of diagnosis and treatment without misdiagnosis.

In addition, there are some limitations in this study. First, this was a retrospective study based on data only from one center and there were certain potential biases. Second, the sample size was small and the follow-up time was short. Thus, in the future, we will further conduct multicenter study with longer follow up to optimize the model. Furthermore, we would prepare for a prospective study and explore other machine learning models.

## Conclusion

In our study, a combined nomogram with clinicopathological variables and ultrasound radiomics signatures was developed and was found to have favorable accuracy for recurrence prediction in PTC patients.

## Data Availability

Data described in the manuscript will be made available upon request pending application and approval from the corresponding author.
